# Synaptic Vesicle Endocytosis in Different Model Systems

**DOI:** 10.3389/fncel.2018.00171

**Published:** 2018-06-28

**Authors:** Quan Gan, Shigeki Watanabe

**Affiliations:** ^1^Department of Cell Biology, Johns Hopkins University School of Medicine, Baltimore, MD, United States; ^2^Solomon H. Snyder Department of Neuroscience, Johns Hopkins University School of Medicine, Baltimore, MD, United States

**Keywords:** synaptic vesicle recycling, synaptic vesicle endocytosis, molecular mechanisms, kinetics of endocytosis, model systems

## Abstract

Neurotransmission in complex animals depends on a choir of functionally distinct synapses releasing neurotransmitters in a highly coordinated manner. During synaptic signaling, vesicles fuse with the plasma membrane to release their contents. The rate of vesicle fusion is high and can exceed the rate at which synaptic vesicles can be re-supplied by distant sources. Thus, local compensatory endocytosis is needed to replenish the synaptic vesicle pools. Over the last four decades, various experimental methods and model systems have been used to study the cellular and molecular mechanisms underlying synaptic vesicle cycle. Clathrin-mediated endocytosis is thought to be the predominant mechanism for synaptic vesicle recycling. However, recent studies suggest significant contribution from other modes of endocytosis, including fast compensatory endocytosis, activity-dependent bulk endocytosis, ultrafast endocytosis, as well as kiss-and-run. Currently, it is not clear whether a universal model of vesicle recycling exist for all types of synapses. It is possible that each synapse type employs a particular mode of endocytosis. Alternatively, multiple modes of endocytosis operate at the same synapse, and the synapse toggles between different modes depending on its activity level. Here we compile review and research articles based on well-characterized model systems: frog neuromuscular junctions, *C. elegans* neuromuscular junctions, *Drosophila* neuromuscular junctions, lamprey reticulospinal giant axons, goldfish retinal ribbon synapses, the calyx of Held, and rodent hippocampal synapses. We will compare these systems in terms of their known modes and kinetics of synaptic vesicle endocytosis, as well as the underlying molecular machineries. We will also provide the future development of this field.

## Introduction

Synaptic vesicle recycling has been studied extensively for over 40 years in various model systems. These studies led to the discovery of five distinguishable mechanisms: clathrin-mediated endocytosis, fast compensatory endocytosis, activity-dependent bulk endocytosis, ultrafast endocytosis, and kiss-and-run (von Gersdorff and Matthews, 1994a; Saheki and De Camilli, 2012; Rizzoli, 2014). The clathrin-mediated endocytosis model proposes that synaptic vesicles fuse with and fully collapse into the plasma membrane; vesicle components then diffuse to endocytic zones and are retrieved via clathrin scaffolds. Likewise, ultrafast endocytosis, fast compensatory endocytosis and bulk endocytosis involve full collapse fusion of synaptic vesicles, but the vesicle components are thought to be retrieved from the plasma membrane via clathrin-independent mechanisms. Synaptic vesicles are then regenerated from the internalized membrane or synaptic endosomes. The most notable features that distinguish these three pathways are the sizes of the endocytic membrane, stimulation conditions, and kinetics. Ultrafast endocytosis generates somewhat uniformly-sized vesicles (diameter of 60–80 nm) within 30−1000 ms of action potentials and occurs with mild stimulation at physiological temperature (Watanabe et al., 2013a,b). Like ultrafast endocytosis, fast compensatory endocytosis is triggered by mild stimulation but is relatively slower (*τ* = 1–2 s; von Gersdorff and Matthews, 1994a; Renden and von Gersdorff, 2007; Soykan et al., 2017). This endocytic pathway is also temperature-sensitive (Renden and von Gersdorff, 2007) and generates endocytic vesicles several times larger than synaptic vesicles (Paillart et al., 2003). Both ultrafast endocytosis and fast compensatory endocytosis retrieve all exocytosed membrane in a short period, and thus both mechanisms are compensatory in nature. In contrast, activity-dependent bulk endocytosis is induced at both physiological and non-physiological temperatures by high-frequency (typically non-physiological) stimulation or application of high potassium (Clayton and Cousin, 2009). This endocytic pathway occurs on a slower time scale (8–20 s; Wu and Wu, 2007) and internalizes large pieces of membrane of random size commensurate to the amount of fusion. Unlike the other four mechanisms, the kiss-and-run endocytosis does not involve full collapse fusion—neurotransmitter is instead thought to be released from a transient fusion pore (He and Wu, 2007). Upon release, the pore closes, and the very same vesicle is retrieved. This mechanism has been well-established in secretory cells such as chromaffin cells (Artalejo et al., 1998; Elhamdani et al., 2006). Several studies have demonstrated the existence of kiss-and-run at conventional synapses, yet its occurrence under physiological conditions remains under extensive debate (He et al., 2006; He and Wu, 2007; Zhang et al., 2009; Wu et al., 2014). Nevertheless, these five mechanisms are thought to be the core recycling pathways for synaptic vesicles.

What factors determine the mode of endocytosis at a particular synapse? At any given presynaptic terminal, one form of endocytosis might predominate. Alternatively, multiple forms of endocytosis might cooperate, depending on the availability of vesicles at the terminals and the activity level, to meet the demand for synaptic vesicle recycling. Here, we review the literature concerning synaptic vesicle endocytosis in a number of well-characterized model systems: frog neuromuscular junctions, *Caenorhabditis elegans (C. elegans)* neuromuscular junctions, *Drosophila* neuromuscular junctions, lamprey reticulospinal giant axons, ribbon synapses of goldfish retinal bipolar neurons, the calyx of Held, and rodent hippocampal synapses. For each synapse, we will first describe its anatomical and functional features, then review what is known about endocytosis and its molecular requirements in each system. Furthermore, we will discuss whether some earlier observations may be explained by ultrafast endocytosis, since this pathway was only discovered in 2013. We will close with perspectives on the future development of this field.

## Frog Neuromuscular Junctions

### Anatomical and Functional Overview

**Figure 1:**
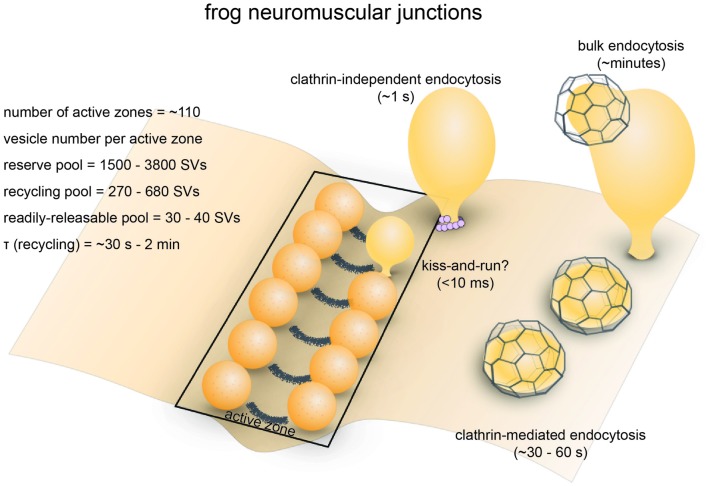
Vesicle pools and endocytic pathways at frog neuromuscular junctions. Frog neuromuscular junctions possess on average 110 active zones. At each active zone, 30–40 vesicles are docked on either side of a central “ridge.” These vesicles represent the readily-releasable pool (RRP). The number of recycling pool vesicles per active zone is 270–680, and the number of reserve pool vesicles per active zone is 1500–3800. During physiological stimulation, vesicle recycling takes place on a time scale of 30 s−2 min. Two endocytic pathways are found at frog neuromuscular junctions: a fast pathway that internalizes vesicles and cisternae within a minute, and a slow pathway that internalizes vesicles at ~8 min during intense stimulation by high K^+^. The fast pathway is mediated by clathrin-independent endocytosis (potentially ultrafast endocytosis or fast compensatory endocytosis) and clathrin-mediated endocytosis. The slow pathway represents activity-dependent bulk endocytosis. An alternative fast pathway kiss-and-run is also suggested. This pathway is predicted to occur within 10 ms. However, the existence of kiss-and-run at frog neuromuscular junctions is still controversial.

The neuromuscular junctions of the sartorius or cutaneous pectoris muscles of frogs (*Rana pipiens*) were the model systems used to study synaptic vesicle dynamics. The anatomical and functional features of these neuromuscular junctions have been well characterized since initial work by Katz and colleagues in the 1950s (Fatt and Katz, 1951, 1952; del Castillo and Katz, 1954, 1956). Sartorius and cutaneous pectoris motor neurons are anatomically similar (Grinnell and Herrera, 1980). Cholinergic motor neuron axons extend elongated unmyelinated branch terminals (total length 100–300 μm; Pawson et al., 1998) running parallel to the muscle fiber (Birks et al., 1960). Fusion sites, or active zones (Couteaux and Pécot-Dechavassine, 1970), are organized in periodic 1.0 μm-long “stripes” running transverse to the terminal, with roughly 0.8–1.1 μm spacing in between (Propst et al., 1986; Pawson et al., 1998). The total number of active zones per neuromuscular junction ranges between 50–250 (110 on average). Along the central axis of each active zone, the plasma membrane curves out to form a 75–90 nm-wide central “ridge” (Figure 1; Heuser et al., 1974; Stoschek et al., 2001). Flanking the ridge on both sides are 30–40 vesicles in physical contact (or “docked”) with the plasma membrane (Couteaux and Pécot-Dechavassine, 1970; Heuser et al., 1979; Pawson et al., 1998). Other vesicles in the terminals also tend to cluster above the active zone (Heuser et al., 1974). Active zones are often directly apposed to 50–100 nm-wide invaginations on the post-junctional membrane of the muscle, known as junction folds (Heuser and Reese, 1973). Acetylcholine receptors are concentrated at the crests of junction folds (Matthews-Bellinger and Salpeter, 1978). This architecture allows close alignment of postsynaptic acetylcholine receptors with presynaptic fusion sites and facilitates efficient activation of the receptors (York and Zheng, 2017).

The presynaptic bouton of frog neuromuscular junctions contains many vesicles, which are divided into functional pools (for definition of pools, see Table 1). There are roughly 200,000 to 500,000 synaptic vesicles at each terminal (Heuser and Reese, 1973). Of these, an estimated 4400 (0.8%–2%) are docked (assuming 40 docked vesicles per active zone and active zones per junction, Pawson et al., 1998). These vesicles likely represent the vesicles in the readily-releasable pool (RRP), since a brief high-frequency stimulation train (30 Hz for 0.5 s triggers fusion of roughly the same number of vesicles (about 2 vesicles per active zone per action potential; Rizzoli and Betz, 2004). In addition, about 15% (30,000–75,000) of vesicles at each terminal constitute the recycling pool. This pool of vesicles sustains synaptic transmission indefinitely under low-frequency stimulation (2–5 Hz) but is depleted at a time constant of 4 s during 30 Hz stimulation, which overwhelms the rate of synaptic vesicle recycling at this terminal (Richards et al., 2003). The rest of the vesicles likely belong to the reserve pool, which is only used during intense, prolonged stimuli. The reserve pool is depleted at a time constant of 40 s during 30 Hz stimulation (Richards et al., 2003). Given the number of vesicles and the rate of fusion, the recycling pool would be consumed completely within 50 s without endocytic processes when these neurons are stimulated at 5 Hz. Thus, the time course of the entire vesicle recycling process cannot be longer than 50 s.

**Table 1:** Definition and characteristics of major synaptic vesicle pools.

	Readily-releasable pool (RRP)	Recycling pool	Reserve pool
Functional definition	Can be consumed by brief stimulation (<1 s); can also be accessed by hypertonic shock	Consumed only after the RRP has been depleted (from <1 s to 1 min)	Released only during intense activity (minutes of high frequency stimulation or high K^+^)
Ultrastructural feature	Docked at or close to the active zone	Close to the active zone membrane; recycled vesicles scattered in the terminal but preferentially utilized	Vesicles found in the cluster typically further away from the plasma membrane
Fraction of the total pool	0.3%–4%, but 0.15% in retinal bipolar neurons	3%–15%; but 0.5% in retinal bipolar neurons	
Mode of replenishment	Does not require recycling; vesicles are readily-available at the active zone.	Endocytosis during mild to moderate stimulation; rate of fusion cannot exceed the kinetics of vesicle regeneration and replenishment; does not require vesicles from the reserve pool	Endocytosis during intense stimulation; likely involves bulk endocytosis; very slow mixing with recycling pool

### Strengths as a Model System

Frog neuromuscular junctions were so widely used in early studies of synaptic vesicle dynamics owing to their numerous advantages as a model system. First, frog cutaneous pectoris or sartorius nerve-muscle preparations are easy to dissect, and the activity at the neuromuscular junctions can be conveniently controlled and monitored *in vitro* (Heuser et al., 1974). Second, active zones and docking sites are highly compartmentalized and distinguishable from endocytic sites, and thus endocytic events can be identified easily using freeze-fracture and ultrathin-section electron microscopy (Heuser and Reese, 1973; Heuser et al., 1974). This organization also results in a very distinct and easily recognizable pattern upon FM dye staining (Betz and Bewick, 1992, 1993). Third, innervated muscles treated with curare can be impaled with a microelectrode so that endplate potentials (EPPs) can be recorded (Betz and Bewick, 1993). Low-dose curare prevents muscle twitching during recordings by attenuating nicotinic acetylcholine receptor-mediated EPPs without affecting presynaptic function (Auerbach and Betz, 1971). Finally, the presynaptic vesicle pool can sustain several minutes of high-frequency activity before it is depleted due to its sheer size (Ceccarelli et al., 1973). These features make it possible to monitor synaptic vesicle dynamics by a combination of electrophysiology, optical imaging and electron microscopy.

### Endocytic Pathways

Optical imaging with lipophilic dyes and electrophysiological recordings suggest the existence of fast and slow pathways at frog neuromuscular junctions (Figure 1). Fei Mao (FM) dyes, which fluoresce strongly when bound to membrane (Gaffield and Betz, 2007), can be taken up via activity-dependent endocytosis (staining) and then released by subsequent stimulus-induced exocytosis (de-staining). Since the mid-1990s, FM dyes have been used to measure the kinetics of synaptic vesicle endocytosis and recycling at frog neuromuscular junctions (Betz and Bewick, 1993; Wu and Betz, 1996). During a train of action potentials (2–30 Hz), the number of vesicle fusion events measured by FM dye de-staining initially matches the number of quanta estimated from EPP recordings. However, after a brief period varying from 30 s to 2 min, EPP size begins to overtake FM dye de-staining, indicating that a population of vesicles that do not contain FM dyes are used (Betz and Bewick, 1993). Presumably, these vesicles are endocytosed after the onset of the action potential train and were fully regenerated and recruited to the RRP within the 30 s–2 min delay period. Thus, a relatively fast pathway for vesicle recycling likely exist at frog neuromuscular junctions. Indeed, following 10 s stimulation at 30 Hz (300 action potentials), endocytosis occurs with a time constant (*τ*) of 23 s (Wu and Betz, 1996). In contrast, after longer trains of stimuli at 30 Hz, the time constant for endocytosis slows down to 57 s following 1800 action potentials and 460 s following 9000 action potentials, suggesting the activation of a slower endocytic pathway by intense activity (Wu and Betz, 1996). A similar study found that following a 1 min tetanus at 30 Hz, both fast and slow endocytic pathways are present at frog muscular junctions (Richards et al., 2000). The slow pathway retrieves membrane with a half-time of about 8 min, and vesicles recycled via this pathway are only available after a rest period of 15–20 min (Richards et al., 2000). FM dye taken up via the fast pathway can be fully released immediately, while FM dye taken up via the slow pathway can only be released after the recycling pool is depleted (Richards et al., 2000). These results suggest that the fast pathway preferentially replenishes the recycling pool while the slow pathway regenerates vesicles in the reserve pool. Thus, the fast and slow pathways are likely to be distinct both in terms of kinetics and molecular mechanisms.

What is the nature of the fast and slow endocytic pathways? Ultrastructural studies suggest that endocytosis occurs via three distinctive mechanisms. Classic experiments by Heuser and Reese (1973) showed that clathrin-coated pits and vesicles as well as cisternae with coated buds accumulate in the terminals following 10 Hz stimulation for 1 min, indicating that this endocytic pathway likely represents the fast pathway observed by FM dye de-staining experiments. They proposed that vesicles are recovered via clathrin-mediated endocytosis and recycled vesicles fuse to form cisternae. Later, they refined this model based on the observation that large pieces of membrane can be directly internalized from the plasma membrane within 1 s following a single action potential and suggested that synaptic vesicles are retrieved through two routes: direct internalization of cisternae and clathrin-mediated endocytosis (Heuser and Reese, 1979; Miller and Heuser, 1984). The fast cisternae uptake pathway may represent either ultrafast endocytosis or fast compensatory endocytosis based on its kinetics and morphology (Heuser and Reese, 1979). These endocytic pathways likely account for the fast recovery route. In contrast, elongated membrane-bound structures (cisternae) primarily form after prolonged intense stimulation (10 Hz, 15 min; Heuser and Reese, 1973). Full recovery from this treatment requires 15–60 min, suggesting that the additional slow pathway likely involve bulk membrane uptake. Thus, a combination of these endocytic pathways could account for both fast and slow endocytic pathways (Figure 1).

Similar experiments by Ceccarelli et al. (1972) led to a slightly different conclusion. After prolonged low-frequency stimulation (2 Hz, 2 h), in two images they observed clathrin-free vesicles containing the fluid phase marker horseradish peroxidase (HRP) associated with the active zone membrane in two different manners. One HRP-containing vesicle was shown to be connected with the plasma membrane via a very narrow neck at the base, while another was in contact with the active zone. This pair of images was interpreted as a vesicle forming a transient fusion pore which then closed (“kiss-and-run”), although it is equally possible that the HRP-containing vesicle was recruited back to the active zone after endocytic recycling. They also observed clathrin-coated pits and vesicles. In a later study from the same group, Torri-Tarelli et al. (1985) followed the ultrastructural change millisecond by millisecond after a single stimulus and noted that the number of fusion intermediates does not increase from 5 ms to 10 ms, while electrophysiological recordings suggest otherwise. Based on these observations, they suggested that some vesicles undergo transient fusion with the membrane (kiss-and-run; Fesce et al., 1994). Though controversial, kiss-and-run may be an alternative fast recycling pathway at the frog neuromuscular junction.

### Molecular Requirements

Frogs were not commonly used for genetic studies until recently (Wang et al., 2015). As a result, the roles of many of the well-known endocytic proteins (e.g., *α*-adaptin, endophilin, Epsin etc.) have not been explored in frog neuromuscular junctions. What little data are available come from studies using chemical probes. For example, the actin polymerization blocker cytochalasin-D prevents the initiation of activity-dependent bulk endocytosis (Nguyen et al., 2012). In contrast, the dynamin GTPase inhibitor Dyngo-4a does not affect the initiation of bulk endocytosis, but traps endocytic intermediates on the cell surface (Nguyen et al., 2012). Analogs of cyclic-GMP (cGMP) accelerate the vesicle cycle at frog neuromuscular junctions, while inhibitors of guanylate cyclase have the opposite effect (Petrov et al., 2008). The accelerating effect of cGMP on the vesicle cycle might be due to an enhancement of the fast endocytic pathway, although direct evidence is lacking. A similar cGMP-dependent regulation of the vesicle cycle mediated by cGKII (cyclic GMP Kinase II) has been found in cerebellar granule cells (Collado-Alsina et al., 2014). In addition, treating motor neurons with methyl-β-cyclodextrin (MβCD) inhibits the uptake of FM1–43 during and after prolonged stimulation, suggesting that membrane cholesterol might play a role in synaptic vesicle endocytosis (Rodrigues et al., 2013). These results, though limited, suggest that synaptic vesicle endocytosis at frog neuromuscular junctions involve complex molecular machineries and are impacted by a wide range of cellular signaling pathways.

## *Caenorhabditis Elegans* Neuromuscular Junctions

### Anatomical and Functional Overview

**Figure 2:**
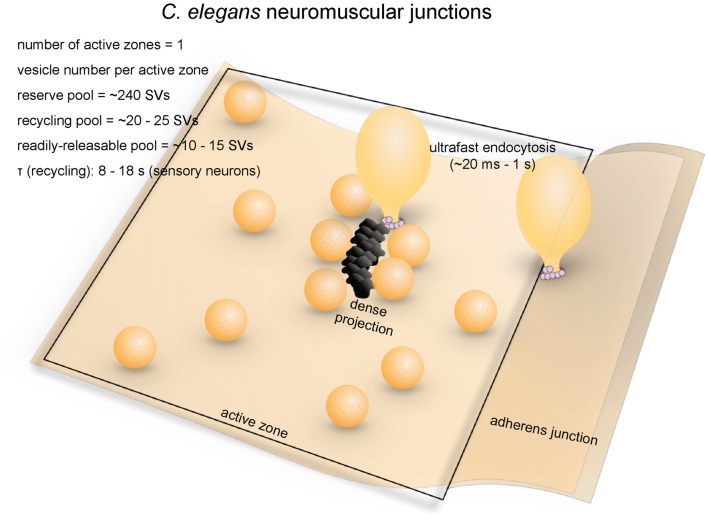
Vesicle pools and endocytic pathways at *C. elegans* neuromuscular junctions. *C. elegans* neuromuscular junctions usually possess a single active zone with a dense projection. An average of 35 vesicles are docked, of which 10–15 can fuse in response to a single stimulus, and therefore represents the RRP. The rest of the docked vesicles (15–20) represent the recycling pool. Roughly 240 vesicles are in the reserve pool. Following single stimuli, ultrafast endocytosis occurs on a time scale of 20 ms–1 s both at the dense projection or at adherens junctions. Bulk endocytosis might also occur at this synapse following hyperstimulation (not shown in figure).

The soil nematode *Caenorhabditis elegans* (*C. elegans*) is an excellent model system for cell biological studies. Adult animals are roughly 1 mm in length and are composed of about 1000 somatic cells, of which exactly 302 are neurons (Jorgensen and Nonet, 1995). The motor neurons of *C. elegans* form *en passant* neuromuscular junctions on ventral and dorsal body wall muscles and innervate muscle cells at multiple locations (Jorgensen and Nonet, 1995). A typical synapse contains a single large active zone with a proteinaceous electron-dense projection (dense projection; Ackermann et al., 2015). The total number of vesicles at a single terminal is roughly 300, of which an average of 34 (11%) are docked (Hammarlund et al., 2007), and these vesicles comprise the recycling pool (Figure 2; Watanabe et al., 2013a). Interestingly, instead of action potentials, graded potentials trigger neurotransmitter release at *C. elegans* neuromuscular junctions (Liu et al., 2009). During sporadic motor neuron activity, the rate of fusion is ~25 vesicles/s per muscle, which is about 1–2 vesicles/s per active zone (Liu et al., 2009). During evoked activity, ~120 vesicles per muscle (~12 vesicles per active zone) fuse in response to each stimulus (Figure 2; Watanabe et al., 2013a). These vesicles presumably represent the RRP.

### Strengths as a Model System

Five features of *C. elegans* allow investigation of vesicle dynamics in molecular detail. First, the nervous system of *C. elegans* is remarkably simple and its synaptic connectivity has been determined by serial-section electron microscopy (White et al., 1986). Second, *C. elegans* colonies are easy to maintain, with a rapid generation cycle of 3 days. In addition, extensive molecular and genetic resources are available and widely shared in the community. Third, the *C. elegans* genome contains single copies of genes, whereas multiple copies are often found in vertebrates (Harris et al., 2001). This lack of genetic redundancy, combined with the surprising viability of the worm without a functional nervous system (Avery and Horvitzt, 1989; Richmond et al., 2001), makes it possible to probe the molecular mechanisms underlying synaptic functions in intact adult animals. Fourth, its small size and optical transparency makes *C. elegans* amenable to optical stimulation and high-pressure freezing experiments (flash-and-freeze). Finally, despite initial technical challenges, protocols for performing whole-cell patch clamping of motor neurons and the muscles of *C. elegans* are now well-developed (Richmond and Jorgensen, 1999). Thus, functional studies can be correlated with ultrastructural analysis in various genetic backgrounds.

### Endocytic Pathways

Little is known about the kinetics of synaptic vesicle recycling at *C. elegans* neuromuscular junctions. Measuring the kinetics using optical methods in motor neurons has been extremely difficult due to autofluorescence from gut granules and refraction of light by the thick cuticle. Based on pHluorin imaging of an odor-responsive sensory neuron, the τ for endocytosis is 8–18 s (Ventimiglia and Bargmann, 2017), which is similar to the time constant of endocytosis in mammalian central synapses (Granseth and Lagnado, 2008; Soykan et al., 2017). This time constant is much faster than the appearance of clathrin-coated pits in frog neuromuscular junctions (Heuser and Reese, 1973; Miller and Heuser, 1984) or the kinetics of clathrin-mediated endocytosis in mammalian cell lines (Kirchhausen et al., 2014). Whether this τ is similar to the time constant in motor neurons is questionable. Nonetheless, a relatively fast mechanism is expected in *C. elegans* neurons.

What are the fast mechanisms that drives synaptic vesicle endocytosis at *C. elegans* neuromuscular junctions? Like in other systems, the main pathway was originally thought to be clathrin-mediated endocytosis (Harris et al., 2001). Consistent with this idea, clathrin adaptor proteins AP2 and AP180 were shown to be required for synaptic vesicle recycling (Nonet et al., 1999). In addition, stonin (UNC-41), which sorts synaptotagmin into the AP2-depedent pathway, contributes to the regeneration of synaptic vesicle pools (Jorgensen et al., 1995; Mullen et al., 2012), suggesting that clathrin-mediated processes are likely essential. However, later studies using more advanced electron microscopy techniques suggested that endocytic processes in* C. elegans* neuromuscular junctions are clathrin-independent.

Recent morphological studies suggest that clathrin-independent ultrafast endocytosis is the primary endocytic pathway in *C. elegans* (Figure 2). Experiments combining optogenetics with high-pressure freezing (flash-and-freeze) of whole animals showed that hyperstimulation (30 s continuous stimulation) of cholinergic neurons resulted in accumulation of endosome-like structures at synapses ~6 s after stimulation, presumably via activity-dependent bulk endocytosis. The diameters of these structures range from 50 nm to 200 nm (100 nm on average). No pits were trapped on the surface even in synaptojanin and endophilin mutants, suggesting that endocytosis can complete without these clathrin-associated proteins (Kittelmann et al., 2013). In the same year, with the finer temporal resolution of the flash-and-freeze approach, another group observed formation of smaller non-coated endocytic vesicles (40 nm on average) adjacent to dense projections within 50 ms or by adherens junctions within 1 s following a single stimulus (Watanabe et al., 2013a). Because of its kinetics, this endocytic pathway was named ultrafast endocytosis. Coated pits and vesicles were not observed in either study, suggesting that clathrin itself may not be essential in synaptic vesicle recycling at these terminals. It is likely that synaptic vesicle endocytosis at *C. elegans* neuromuscular junctions is primarily mediated by ultrafast endocytosis.

### Molecular Requirements

Dynamin is essential for synaptic vesicle recycling at *C. elegans* neuromuscular junctions. A temperature-sensitive mutant similar to the *Drosophila* *shibire*^ts^ mutant, *dyn-1(ky51)*, displays severe locomotion defects (Clark et al., 1997). Following optogenetic hyperstimulation, the neuromuscular junction boutons of *dyn-1* mutants contain large membrane involutions that are continuous with the plasma membrane, suggesting that dynamin is involved in fission of bulk endocytic intermediates (Kittelmann et al., 2013). Occasionally, synaptic vesicle-sized buds formed on these endocytic intermediates. However, the budding of synaptic vesicles from these large vesicles is also blocked in the mutant (Kittelmann et al., 2013). Similarly, *dyn-1(ky51)* displays large vesicles linked to the plasma membrane by narrow necks close to the dense projection 1 s after a single stimulation pulse, indicating a defect in ultrafast endocytosis (Watanabe et al., 2013a). These results highlight the essential role of dynamin in clathrin-independent endocytosis at *C. elegans* neuromuscular junctions.

Clathrin-associated proteins are essential in synaptic vesicle recycling at *C. elegans* neuromuscular junctions. However, it is unclear whether they function on the plasma membrane or at endosomes. Complete loss of the AP2 complex subunits leads to a significant reduction in the number of synaptic vesicles (~70% reduction) and an accumulation of endosomes (Gu et al., 2008; Mullen et al., 2012). Similarly, AP180 is not essential for endocytosis, but regulates the size and protein compositions of synaptic vesicles (Nonet et al., 1999). These results are consistent with the notion that clathrin-mediated endocytosis is not required for membrane retrieval from the cell surface but for the resolution of endosomes. However, synaptic vesicle markers diffusively localize on the plasma membrane in these mutants (Nonet et al., 1999; Mullen et al., 2012), suggesting that adaptor proteins may also function at the plasma membrane to sort vesicular proteins. Likewise, homologs of the clathrin-associated proteins Esp15 (EHS-1) and intersectin-1 (ITSN-1) contribute to synaptic vesicle regeneration (Salcini et al., 2001; Wang et al., 2008). Paradoxically, loss of EHS-1 leads to complete depletion of synaptic vesicles without apparent formation of endosomes, suggesting its function at the plasma membrane, while loss of ITSN-1 results in accumulation of large vesicles in the terminals (Salcini et al., 2001). The amphiphysin and syndapin homologs AMPH-1 and SDPN-1 act on early endosomes to regulate cargo recycling (Pant et al., 2009; Gleason et al., 2016). They are therefore not likely to be directly involved in synaptic vesicle endocytosis from the plasma membrane, although their exact roles are yet to be determined. Thus, although these proteins all interact with clathrin, their functional domains do not necessarily overlap. This suggests that either clathrin-mediated processes occur both at the plasma membrane and endosomes or these proteins have clathrin-independent functions. Further investigation is required to resolve these apparent contradictions.

Similarly, mutations in endophilin (unc-57) and synaptojanin (unc-26) block synaptic recycling in *C. elegans* (Harris et al., 2000; Schuske et al., 2003). Initial experiments using conventional chemical fixation at 4°C showed accumulation of both coated and non-coated pits on the membrane (Schuske et al., 2003). However, later studies employing high-pressure freezing at room temperature reported that endosomes accumulate in the terminals (Kittelmann et al., 2013). This temperature effect is consistent with the observation in mouse hippocampal synapses that ultrafast endocytosis fails at lower temperatures (Watanabe et al., 2014). Thus, clathrin-mediated endocytosis may be able to compensate for the loss of ultrafast endocytosis in *C. elegans* neuromuscular junctions.

While clathrin-associated proteins are essential in *C. elegans* neuromuscular junctions, clathrin itself may not be involved in synaptic vesicle recycling. In mutants with a temperature-sensitive allele of clathrin heavy chain (CHC), shifting to the non-permissive temperature removes almost all CHC but does not significantly reduce the number of synaptic vesicles at steady state or the amplitude of postsynaptic miniature currents (Sato et al., 2009). However, the diameter of synaptic vesicles is smaller, suggesting that clathrin is likely needed to maintain the size, but not the number, of synaptic vesicles. Nevertheless, the hypomorphic nature of this temperature-sensitive mutant means that the residual amount of clathrin might be sufficient to maintain synaptic vesicle recycling. Experiments using a conditional null background will be needed to resolve this uncertainty.

## *Drosophila* Neuromuscular Junctions

### Anatomical and Functional Overview

**Figure 3:**
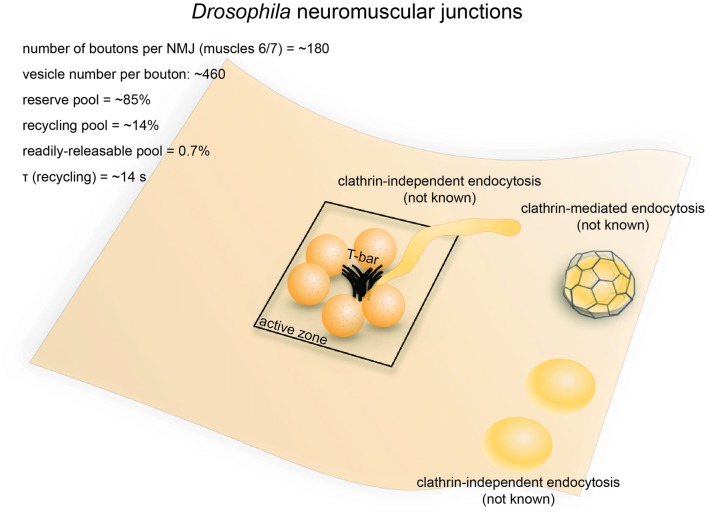
Vesicle pools and endocytic pathways at *Drosophila* neuromuscular junctions. *Drosophila* neuromuscular junctions possess 200–300 active zones. The center of each active zone is marked by a dense projection commonly referred to as a “T-bar”, around which synaptic vesicles tend to cluster. In each bouton, 0.7% of the vesicles on average are readily releasable; ~14% of the vesicles are in the recycling pool; and roughly ~85% are in the reserve pool. Synaptic vesicle endocytosis occurs with a time constant of 14 s as measured by pHluorin. Both clathrin-mediated and clathrin-independent endocytosis occurs at *Drosophila* neuromuscular junctions. One particular form of clathrin-independent endocytosis might occur adjacent to the T-bar. The exact time courses of these endocytic pathways have not been determined.

The neuromuscular junction of fruit flies (*Drosophila*) is an excellent model for studying the synaptic development and function (Keshishian et al., 1996). Like excitatory synapses in the mammalian central nervous system, *Drosophila* neuromuscular junctions use L-glutamate as the neurotransmitter (Jan and Jan, 1976). They have large presynaptic boutons (type Is boutons, ~1–3 μm in diameter; type Ib boutons, ~2–5 μm in diameter (Atwood et al., 1993), with each neuromuscular junction containing roughly ~180 boutons (Schuster et al., 1996), and each bouton containing 7–41 active zones (Atwood et al., 1993). The center of the active zone is marked with a dense projection known as the “T-bar” (due to its apparent shape in electron micrographs from chemically-fixed specimens; Figure 3). The area around the T-bar is enriched in synaptic vesicles. On average, the total number of synaptic vesicles at a single bouton is around 460 (assuming 83,000 vesicles/neuromuscular junction, Delgado et al., 2000) in muscles 6/7 synapse and 180 boutons per neuromuscular junction in muscles 6/7 (Schuster et al., 1996). Of these, roughly 0.7% are in the RRP (Müller et al., 2012), ~14% are in the recycling pool (Delgado et al., 2000) and the rest constitute the reserve pool (Delgado et al., 2000). FM dye staining/de-staining experiments suggest that the recycling pool is located in the periphery of the bouton while the reserve pool resides primarily in the core region (Ramaswami et al., 1994; Kuromi and Kidokoro, 1998). The maximal rate of synaptic vesicle recycling at a typical neuromuscular junction has been estimated to be around 1000/s (3–5/s per active zone; Delgado et al., 2000), implicating that the recycling pool could be completely turned over in roughly 10 s.

### Strengths as a Model System

*Drosophila* neuromuscular junctions are conducive to optical, electrophysiological and genetic approaches. Optical imaging using pHluorin and FM dyes can be performed on *Drosophila* larva, allowing functional measurement of endocytic processes. Post-synaptic responses can be measured with relative ease by performing two-electrode voltage clamp on innervated muscles. Most of the synaptic proteins implicated in mammalian synaptic transmission are well-conserved in *Drosophila* and are expressed at the neuromuscular junction. Methods for genetic manipulations are well-developed, including an extensive toolkit of GAL4/UAS targeted expression lines. These advantages have made the *Drosophila* neuromuscular junction one of the most popular model systems for studying molecular mechanisms of synaptic vesicle endocytosis.

### Endocytic Pathways

The time constant for synaptic vesicle recycling has been determined by optical imaging in *Drosophila* neuromuscular junctions. In wild-type flies, pHluorin recovery following 50 Hz stimulation for 10 s has a time constant of ~14 s (Poskanzer et al., 2006). Similarly, FM dye de-staining experiments suggested a similar time constant for the synaptic vesicle cycle (Delgado et al., 2000). Thus, like in *C. elegans* neurons, a relatively fast mechanism is expected at *Drosophila* neuromuscular junctions.

The mechanisms underlying synaptic vesicle recycling at *Drosophila* neuromuscular junctions have been extensively studied since the discovery of the *shibire*^ts^ (*shi*^ts1^) mutation (Poodry et al., 1973). *Shibire*^ts^ inhibits the function of dynamin in a temperature-dependent manner (Chen et al., 1991; van der Bliek and Meyerowrtz, 1991), thereby stalling endocytosis and allowing endocytic intermediates to be captured by electron microscopy (Kosaka and Ikeda, 1983). In *shi*^ts^ flies, synaptic vesicles in the bouton are depleted under non-permissive temperatures (30°C, 5−10 min), with both coated and uncoated endocytic intermediates stalled throughout the plasma membrane, although the number of coated pits was noted to be low (data not quantified; Kosaka and Ikeda, 1983). Nonetheless, these results suggest the involvement of both clathrin-dependent and clathrin-independent mechanisms in synaptic vesicle recycling at these synapses (Figure 3). Another study in the *shi*^ts^ mutant revealed the possible existence of two separate endocytic pathways at retinula cell terminals: a faster pathway occurring directly at the dense projection, and a slower pathway occurring away from the active zone (Koenig and Ikeda, 1996; Kidokoro, 2006). A similar distinction supposedly exists at neuromuscular junction terminals, given that endocytic structures are trapped at dense projections as well as peri-active zone membrane (Kosaka and Ikeda, 1983). It is possible that the earlier pathway at the active zone represents ultrafast endocytosis or fast compensatory endocytosis, while the later pathways represent clathrin-mediated endocytosis and activity-dependent bulk endocytosis (Figure 3). However, due to the limited temporal resolution associated with chemical fixation, the exact time course of these pathways could not be determined. Nevertheless, the *shi*^ts^ mutant shows synaptic fatigue within 20 ms of repetitive stimulation at non-permissive temperature while wild type flies maintain their synaptic transmission (Kawasaki et al., 2000). This effect is not due to the availability of release-ready vesicles, implying that a fast dynamin-mediated process is required to clear fusion sites for incoming vesicles. Since no evidence has been found to support the existence of kiss-and-run at these synapses (Dickman et al., 2005), such a fast process probably represents ultrafast endocytosis. Future studies using rapid freezing techniques might help resolve this issue.

### Molecular Requirements

The genetic tractability of *Drosophila* makes it an ideal model organism for investigating the molecular requirements of vesicle recycling. As mentioned above, dynamin is required for vesicle recycling at *Drosophila* neuromuscular junctions (Kosaka and Ikeda, 1983). Clathrin and α-adaptin (a component of the AP2 complex) are also both required for vesicle recycling (González-Gaitán and Jäckle, 1997; Heerssen et al., 2008), although it is unclear whether they are directly needed for endocytosis from the plasma membrane. Photoinactivation of clathrin leads to a failure of synaptic vesicle recycling at these terminals, but FM dye can be loaded into the terminals by activity (Heerssen et al., 2008). Similarly, mutation of the *stoned* proteins (Stoned A and Stoned B), which are hubs for Synaptotagmin-1 (Syt I) and AP2, leads to accumulation of fully internalized large endocytic vesicles and a reduced density of synaptic vesicles (Fergestad et al., 1999). Similarly, point mutations in the poly-lysine motif of Syt I, which binds the AP2 complex, results in an accumulation of large vesicles without affecting the rate of endocytosis (Poskanzer et al., 2006). Likewise, null mutation of *syt I* and rapid photoinactivation of Syt I both lead to defect in endocytosis (Poskanzer et al., 2003), suggesting that Syt I, a calcium sensor for exocytosis, may also play a role in endocytosis (Poskanzer et al., 2003). This endocytic function of Syt I seems to depend on its ability to bind calcium, since point mutations in the C2B, but not C2A, domain of Syt1 result in slowed endocytic rate (Poskanzer et al., 2006). These results suggest that clathrin and associated proteins are needed for regeneration of vesicles from endosomes, but not for endocytosis at the plasma membrane, and that calcium may control the rate of endocytosis at the plasma membrane.

The clathrin-associated proteins Eps15 (an EH domain adaptor-like protein) and intersectin-1/Dap60 (Koh et al., 2004; Majumdar et al., 2006) are also required for synaptic vesicle recycling. Mutations in Eps15 lead to defects in FM dye uptake and an accumulation of membrane invaginations, suggesting that it functions at the plasma membrane (Majumdar et al., 2006). This phenotype is rescued by an Eps15 construct lacking its α-adaptin-interacting domain (Koh et al., 2007), suggesting that Eps15 might be involved in clathrin-independent endocytosis.

Several membrane-remodeling proteins are required for synaptic vesicle recycling at *Drosophila* neuromuscular junctions. Mutations in the BAR domain protein endophilin A, which senses and modifies membrane curvature, lead to synaptic vesicle depletion and an accumulation of early endocytic intermediates (Guichet et al., 2002; Rikhy et al., 2002). LRRK2, which regulates the phosphorylation cycle of endophilin A, is also required for normal synaptic vesicle endocytosis (Matta et al., 2012). The polyphosphoinositide phosphatase synaptojanin-1 is recruited to endocytosed vesicles at *Drosophila* neuromuscular junctions and mediates the uncoating of those vesicles (Verstreken et al., 2003). Interestingly, the N-BAR domain protein amphiphysin and the F-BAR domain protein syndapin, both of which participate in clathrin-mediated endocytosis (Takei et al., 1999; Qualmann and Kelly, 2000), are not strictly required for synaptic vesicle endocytosis at *Drosophila* neuromuscular junctions (Razzaq et al., 2001; Kumar et al., 2009). Thus, these membrane-modifying proteins seem to play differential roles in synaptic vesicle recycling.

## Lamprey Reticulospinal Synapses

### Anatomical and Functional Overview

**Figure 4:**
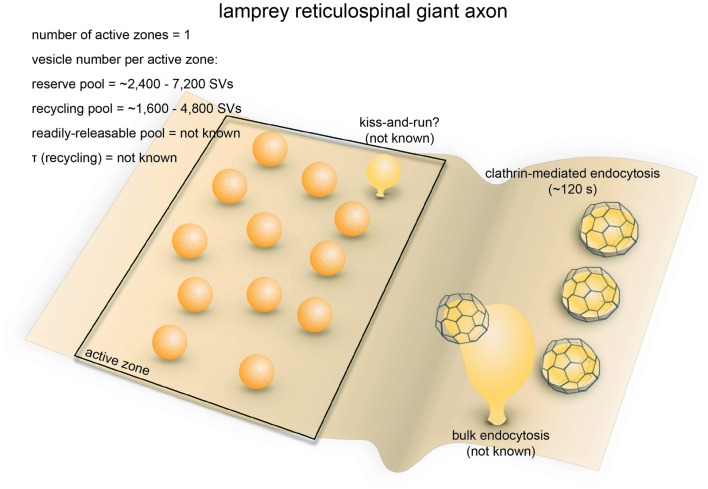
Vesicle pools and endocytic pathways at lamprey reticulospinal giant synapses. Lamprey reticulospinal giant synapses usually possess only one active zone. The exact number of vesicles in the RRP is unknown. Vesicles at this synapse can be divided into a synapsin-independent (recycling) pool of 1600–4800 vesicles and a synapsin-dependent (reserve) pool of 2400–7200 vesicles. The exact time course of vesicle recycling has not been measured. Clathrin-mediated endocytosis occur 40–120 s following prolonged stimulation. Activity-dependent bulk endocytosis also occur. Kiss-and-run has been suggested, but direct morphological evidence is lacking.

The nervous system of lampreys, a class of jawless fish belonging to the order *Petromyzontiformes*, represents the most primitive form among vertebrates (Xu et al., 2016). The giant axons of reticulospinal neurons (also known as Müller cells) in adult lampreys (usually *Lampetra fluviatilis* or *Petromyzon marinus*) can reach up to 80 μm in diameter. These axons are specialized in transmitting phasic signals by firing brief high-frequency spike trains (usually lasting less than 2 s; Brodin et al., 1997, 1999), with maximal firing frequencies in the range of 20–30 Hz (average 4.4 ± 4.9 Hz; Zelenin, 2011). They form *en passant* chemical (glutamatergic) and electrical synapses on the dendrites of spinal neurons in the lateral column (Vesselkin et al., 1995; Vinay et al., 1998). Most of the synapses have a single oval-shaped active zone (simple synapses) while a fraction of them have 2–3 active zones (complex synapses). For simple synapses, the diameter of the active zone ranges from 0.8 μm to 1.8 μm (1.2 μm on average; Gustafsson et al., 2002). The number of synaptic vesicles per synapse ranges from 4000 to 12,000 depending on active zone area (Figure 4; Gustafsson et al., 2002). Vesicles can be divided into two separate pools: a synapsin-independent proximal pool immediately adjacent to the active zone membrane and a synapsin-dependent distal pool residing >200 nm from the plasma membrane (Pieribone et al., 1995). The synapsin-dependent pool contains ~60% of all vesicles within 500 nm of the active zone and is thought to represent the reserve pool, while the synapsin-independent pool represents vesicles in the readily-releasable and recycling pools (Pieribone et al., 1995; Vesselkin et al., 1995).

### Strengths as a Model System

Vesicle dynamics have been studied extensively in reticulospinal giant synapses in lampreys mainly for the following four reasons (Rovainen, 1974). First, the remarkable size of lamprey reticulospinal axons permits microinjection of antibodies and active peptides into the cytoplasm to sequester and disrupt specific molecular targets (Gustafsson et al., 2002; Brodin and Shupliakov, 2006). This approach compensates for the lack of genetic manipulation tools in lampreys. Second, the active zones of reticulospinal synapses are well-separated from each other, making it possible to study vesicle dynamics at individual active zones (Gustafsson et al., 2002). Third, by microinjecting Ca^2+^-sensitive dyes into the axons, Ca^2+^ influx at individual active zones can be monitored over time (Brodin and Shupliakov, 2006). Fourth, action potentials in reticulospinal axons as well as postsynaptic currents in the lateral column cells they innervate can be conveniently recorded using intracellular or extracellular electrodes (Brodin et al., 1994). The reticulospinal giant synapses are thus a convenient model for studying the synaptic vesicle cycle in vertebrates.

### Endocytic Pathways

Synaptic vesicle recycling at lamprey reticulospinal synapses is mainly governed by clathrin-mediated endocytosis (Figure 4). Time constants for endocytosis at these synapses have not been measured using optical imaging approaches. Instead, time-resolved electron microscopy has been employed to monitor the endocytic events. Prolonged high-frequency stimulation (20–30 Hz, 15–30 min) causes clathrin-coated pits to appear on the plasma membrane (Kershaw and Christensen, 1980). The formation of these coated pits is blocked by placing the spinal cord in a Ca^2+^-free solution immediately after stimulation (20 Hz, 20 min) but resumes upon re-exposure to Ca^2+^ (Gad et al., 1998). Based on the increase in the number of coated pits over time after Ca^2+^ re-exposure, the rate of vesicle recycling through clathrin-mediated endocytosis is around 0.4 vesicles/s per synapse (Gad et al., 1998). Most of the coated pits are shallow at the 40 s time point following Ca^2+^ re-exposure, while they are deeply invaginated by the 120 s time point. The kinetics of coated pit maturation is similar to that of the receptor-mediated endocytosis (Kirchhausen et al., 2014), suggesting that lamprey reticulospinal synapses employ slow clathrin-mediated endocytosis as the primary route for synaptic vesicle endocytosis.

In addition to coated pits, plasma membrane invaginations and endosome-like vacuoles connected to the plasma membrane also emerge following prolonged stimulation. Clathrin-coated pits occasionally form at the tip of these deep invaginations (Figure 4; Gad et al., 1998). These observations are consistent with the presence of activity-dependent bulk endocytosis. Thus, in addition to clathrin-mediated endocytosis, synaptic vesicle recycling at lamprey reticulospinal synapses involves activity-dependent bulk endocytosis.

In contrast, little work in lamprey has explored fast endocytosis. The large reserve vesicle pool per active zone, combined with the phasic activity pattern, likely results in a low demand on vesicle recycling kinetics at reticulospinal synapses (Brodin et al., 1999). Based on the modulation of quantal size by serotonin, a role has been proposed for kiss-and-run (Photowala et al., 2006), although direct morphological evidence is still lacking. No ultrastructural evidence has been found for ultrafast endocytosis. However, this could be due to the limited temporal resolution of existing studies or the stimulation paradigms used. Almost all electron microscopy studies are performed following prolonged high-frequency stimulation, lasting 15–30 min, whereas intrinsic activity pattern is phasic (0.3–34 Hz, lasting seconds; Zelenin, 2011). How endocytosis takes place under a more physiological activity pattern has not been explored in this system.

### Molecular Requirements

Since genetic approaches are not possible in lamprey, molecular mechanisms are probed by microinjection of antibodies and small peptides against candidate proteins implicated in clathrin-mediated endocytosis (Brodin and Shupliakov, 2006). These antibodies and peptides specifically disrupt protein-protein interactions. Following application, clathrin-mediated endocytosis is perturbed at various stages, indicating the step at which each protein acts. For example, disrupting the intersectin-AP2 interaction leads to an accumulation of shallow coated pits, implying a block at the early stage of clathrin-mediated endocytosis (Pechstein et al., 2010). Microinjecting Epsin1 antibodies yields a similar phenotype (Jakobsson et al., 2008), suggesting that Epsin1 is also an early factor. Similarly, disrupting the CLAP domain-mediated interaction between amphiphysin and clathrin depletes the vesicle pool and causes accumulation of coated pits that are abnormally-shaped (Evergren et al., 2004), suggesting that amphiphysin plays an essential role in proper maturation of clathrin-coated pits. Furthermore, disrupting the SH3 domain on amphiphysin leads to a depletion of synaptic vesicles and an accumulation of clathrin-coated pits with a narrow neck, suggesting that the SH3 domain of amphiphysin is needed to recruit dynamin (Shupliakov et al., 1997). Likewise, disrupting the function of the SH3 domain of endophilin causes accumulation of clathrin-coated intermediates with a narrow neck as well as coated vesicle that are fully internalized (Ringstad et al., 1999; Gad et al., 2000). The accumulation of clathrin-coated pits likely results from the endophilin failing to recruit dynamin, which may be compensated by other proteins like amphiphysin (Sundborger et al., 2011). On the other hand, accumulation of coated vesicles is likely due to a loss of endophilin-synaptojanin interaction, since synaptojanin is essential for uncoating (Gad et al., 2000). Thus, proteins associated with clathrin-mediated endocytosis form a complex network of interactions and cooperatively promote the formation and maturation of clathrin-coated vesicles.

The actin cytoskeleton also contributes to clathrin-mediated synaptic vesicle recycling in lamprey. Filamentous actin (F-actin) is organized in a donut-shape at lamprey reticulospinal synapses, presumably surrounding the active zone (Shupliakov et al., 2002). Stimulation increases F-actin signal in the adjacent endocytic zone (Shupliakov et al., 2002). Acutely disrupting actin polymerization leads to an accumulation of coated pits with a wide-open neck at the plasma membrane, whereas blocking actin depolymerization causes recycled vesicles to be trapped between the endocytic site and the active zone vesicle cluster (Shupliakov et al., 2002). These results indicate that actin dynamics are involved at multiple stages of the vesicle recycling process at reticulospinal synapses.

In addition to clathrin-mediated endocytosis, activity-dependent bulk endocytosis operates under high frequency stimulation; this process is, in part, mediated by syndapin. Microinjecting an antibody against the SH3 domain of lamprey syndapin leads to no detectable defect in vesicle recycling following low-frequency stimulation (Andersson et al., 2008). However, under intense stimulation, disrupting syndapin function leads to a decrease in vesicle number and an accumulation of large VAMP-containing cisternae, some of which are connected to the plasma membrane (Andersson et al., 2008). These results suggest that syndapin is not strictly required for clathrin-mediated endocytosis, but may help regenerate synaptic vesicles from membrane intermediates during activity-dependent bulk endocytosis. In addition, adding excessive amounts of synuclein, a protein implicated in Parkinson’s disease that regulates synaptic vesicle endocytosis at mammalian synapses (Lautenschläger et al., 2017), depletes synaptic vesicles and causes cisternae and clathrin-coated pits to accumulate under intense stimulation (20 Hz, 5 min; Busch et al., 2014; Medeiros et al., 2017). These results indicate that synuclein plays a regulatory role in clathrin-mediated endocytosis and activity-dependent bulk endocytosis at lamprey reticulospinal synapses.

## Ribbon Synapses of Retinal Bipolar Neurons

### Anatomical and Functional Overview

**Figure 5:**
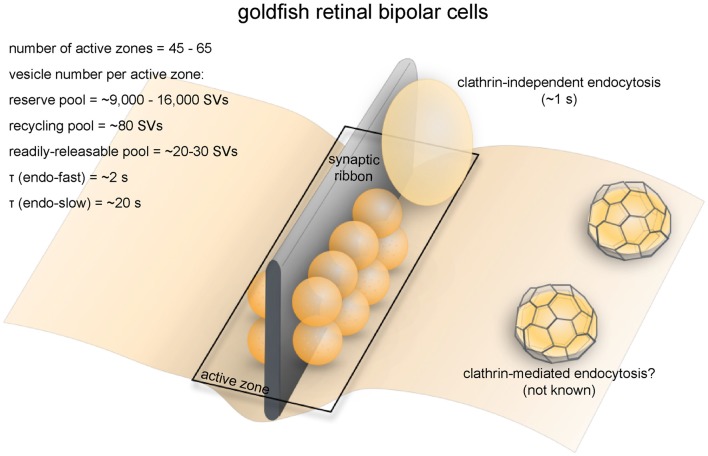
Vesicle pools and endocytic pathways at goldfish bipolar neuron terminals. The axonal terminals of goldfish bipolar neurons possess 45–65 active zones. At each active zone, there is a large electron-dense body known as the synaptic ribbon. Twenty to 30 vesicles are docked at the base of the synaptic ribbon and are readily releasable. Another 80 or so are tethered to the ribbon; these vesicles belong to the recycling pool. Nine-thousand to 16,000 vesicles per active zone are in the reserve pool. Two endocytic pathways exist at these terminals: a fast pathway (*τ*_fast_ ≈ 2 s) following weak stimulation and a slow pathway (*τ*_slow_ ≈ 20 s with delayed initiation) following repeated strong stimulation. The fast pathway is likely operated by clathrin-independent endocytosis (potentially ultrafast endocytosis or fast compensatory endocytosis), while the slow pathway could represent either clathrin-mediated endocytosis or activity-dependent bulk endocytosis.

Ribbon synapses are found in vertebrate sensory systems that use graded potentials instead of action potentials for neurotransmission (Sterling and Matthews, 2005). They are characterized by a striking ultrastructural feature: the synaptic ribbon, which is typically a 30 nm-thick plate jutting ~200 nm from each active zone into the cytoplasm (Figure 5). Prominent examples of ribbon synapses include those of photoreceptors, retinal bipolar neurons, auditory or vestibular hair cells, and electrosensory receptors (Sterling and Matthews, 2005). These synapses are anatomically and functionally diverse, although they invariably use L-glutamate as the primary neurotransmitter (Sterling and Matthews, 2005). For the sake of simplicity, here we will focus on the ribbon synapses of goldfish retinal bipolar cells.

Retinal bipolar cells relay information from photoreceptors to ganglion cells and amacrine cells. Goldfish Mb1-type bipolar cells have large bulbous axonal terminals (8–12 μm in diameter; Lagnado et al., 1996; von Gersdorff and Matthews, 1999), which makes them a very popular system for studying the synaptic vesicle dynamics of ribbon synapses (Figure 5). Each terminal contains 45–65 ribbons (von Gersdorff et al., 1996). The total number of vesicles per terminal is estimated to range from ~500,000 to 900,000 depending on the size of the terminal (von Gersdorff et al., 1996). Among these vesicles, 1000–1800 (0.15%–0.3%) belong to the RRP while 4500 (0.5%–0.9%) belong to the recycling pool, adding up to about 5500 vesicles that are available for depolarization-evoked fusion (Neves and Lagnado, 1999; von Gersdorff and Matthews, 1999). This number corresponds well with the ultrastructural observation that at each terminal roughly 6000 vesicles on average are tethered to ribbons, and of these about 1200 are docked (22 per active zone; von Gersdorff et al., 1996).

Capacitance measurements and FM dye imaging indicate that vesicle fusion at ribbon synapses occurs at exceptionally high rates (von Gersdorff et al., 1996). In response to depolarizing pulses, vesicles of the RRP are depleted within 20 ms (Neves and Lagnado, 1999), giving a fusion rate of 900–1600/s per active zone. The recycling pool is depleted within 200 ms (von Gersdorff and Matthews, 1999), giving a fusion rate of 410/s per active zone. Vesicles can also fuse continuously at low intracellular calcium concentrations without any apparent depletion (Lagnado et al., 1996; Rouze and Schwartz, 1998). This continuous vesicle cycle entails balanced exo- and endocytosis at a maximal rate of 900 vesicles/s (Rouze and Schwartz, 1998; or 14–20/s per active zone), equivalent to the turnover of the entire surface area of the terminal every 2 min. Another study found that with sustained depolarization using 50 mM K^+^ at 2.5 mM Ca^2+^, the continuous recycling rate can be as high as 3800/s (Lagnado et al., 1996; or 69/s per active zone). In contrast, conventional synapses between amacrine cells have a maximal fusion rate of roughly 20/s per active zone (Stevens and Tsujimoto, 1995). Thus, functional properties of synaptic transmission are well characterized, making these neurons ideal model systems for studying synaptic vesicle recycling in synapses that engage in high-rate graded neurotransmission.

### Strengths as a Model System

Axonal terminals of goldfish bipolar neurons possess unique advantages for studying synaptic vesicle dynamics. Bipolar neurons can be easily extracted from dissociated retinal tissue. Due to their large size, high fusion rate and simple morphology, bipolar neuron terminals are well-suited for direct measurements of membrane capacitance changes (Mennerick et al., 1997). Whole-cell patch clamp recording can be performed either on the cell body or directly on the synaptic bouton. A sinusoidal voltage can be superimposed on the holding potential, and the resulting current can be analyzed to derive the time-resolved membrane capacitance of the cell (von Gersdorff and Matthews, 1999). The change in membrane capacitance following the fusion of a single vesicle roughly equals to the capacitance of that vesicle (roughly 3 × 10^−17^ F; Matthews, 1999; von Gersdorff and Matthews, 1999). Due to the small size of this change, exo- and endocytosis can only be accurately assayed by capacitance measurement at synapses where the rates of these processes are very high (Matthews, 1999). The advantage of capacitance measurement over other electrophysiological approaches and pHluorin imaging is that it can monitor membrane flux independent of other steps of the vesicle cycle such as endosomal sorting and reacidification. In addition, the superior time resolution of capacitance measurement makes it more suitable at determining the kinetics of exo- and endocytosis than pHluorin and FM dye imaging studies. Although goldfish is not conventionally used for genetic studies, molecular mechanisms of the synaptic vesicle cycle can still be investigated in goldfish bipolar neurons using purified protein domains and antibodies in the internal pipette solution during patch clamp experiments.

### Endocytic Pathways

Membrane capacitance measurements revealed two kinetically distinct endocytic pathways at bipolar neuron ribbon synapses (Figure 5; von Gersdorff and Matthews, 1994a). In response to a single 250 ms depolarizing pulse, membrane capacitance rapidly increases due to vesicle fusion, followed by fast recovery back to the original level due to compensatory endocytosis at a time constant (τ) of 2 s at room temprautre (von Gersdorff and Matthews, 1994a,b). Capacitance recovery following a weaker (10 ms) stimulus has a similar rate (*τ* = 1.4 s; von Gersdorff and Matthews, 1994b). In contrast, after a train of strong stimuli, capacitance recovery initiates 2 s later (von Gersdorff and Matthews, 1994b) and proceeds much more slowly (τ ≈ 20 s; von Gersdorff and Matthews, 1994a,b). The slow pathway might be recruited due to the fast pathway being inhibited by high intracellular [Ca^2+^] (von Gersdorff and Matthews, 1994b) during strong stimuli (Llobet et al., 2011). Endocytosis is also slowed by high [Ca^2+^]_i_ at the ribbon synapses of mouse rod bipolar cells (Wan et al., 2008). A similar phenomenon is found in conventional central synapses (Leitz and Kavalali, 2011; Armbruster et al., 2013; see later sections), but might only be physiologically significant at synapses that normally experience sustained high-frequency activity.

The fast pathway of membrane retrieval at bipolar neuron terminals is operated by fast compensatory endocytosis (Figure 5). In fact, bipolar neuron ribbon synapses are the model system where this form of endocytosis was originally discovered (von Gersdorff and Matthews, 1994a). FM dye imaging and interference reflection microscopy studies indicate that synaptic vesicles fully collapse during exocytosis at retinal bipolar neuron terminals (Zenisek et al., 2002; Llobet et al., 2003), thereby precluding kiss-and-run as an alternative for the fast pathway. Non-coated large vesicles several times the size of a synaptic vesicle are observed at bipolar terminals either during spontaneous calcium spiking or after a brief high-[K^+^]_o_ pulse (Paillart et al., 2003). The shape of endocytosed vesicles is similar to ultrafast endocytic intermediates (Watanabe et al., 2013b). However, the fast pathway at bipolar neuron terminals has slower kinetics (1–2 s) than ultrafast endocytosis (50–1000 ms) at conventional synapses (Watanabe et al., 2013b). Moreover, disrupting F-actin, which is essential for ultrafast endocytosis, does not affect the fast component of endocytosis (Holt et al., 2003), suggesting that this fast pathway may be driven by a mechanism distinct from ultrafast endocytosis. Nevertheless, detailed temporal analysis is likely required to determine the exact nature of this fast pathway.

The slow pathway is most likely to be operated by activity-dependent bulk endocytosis (Figure 5), given that both appear under sustained stimulation. The presence of large “vacuoles” near the sites of exocytosis further supports this conclusion (Holt et al., 2003). Bulk endocytosis is also observed at the ribbon synapses of frog saccular hair cells (Lenzi et al., 2002). In addition to bulk membrane uptake, clathrin-mediated endocytosis may also contribute to the slow pathway, as FM dyes are taken up into small vesicles (Holt et al., 2003). However, clathrin-coated vesicles are rarely observed at goldfish bipolar neuron terminals (Paillart et al., 2003; LoGiudice and Matthews, 2007), questioning whether those small vesicles represent endocytic intermediates from the clathrin-mediated pathway. Interestingly, at the finely-branched axon terminals of mouse retinal bipolar neurons, prolonged stimulation by high [K^+^]_o_ at room temperature triggers clathrin-mediated endocytosis instead, with little or no sign of bulk retrieval (LoGiudice et al., 2009). It is not clear what underlies the difference between mammalian retinal bipolar neurons and those of other vertebrates.

### Molecular Requirements

Proteins implicated in clathrin-mediated endocytosis are involved in vesicle recycling at retinal bipolar neuron terminals. Both dynamin and clathrin are found in the inner plexiform layer of the goldfish retina, where the ribbon synapses of bipolar neurons are located (Sherry and Heidelberger, 2005). Intracellular dialysis of a non-hydrolyzable GTP analog, GTP-γ-S, inhibits both the fast and slow components of synaptic vesicle endocytosis (Jockusch et al., 2005), indicating that dynamin is involved in vesicle retrieval from the plasma membrane. On the other hand, intracellular dialysis of the clathrin- and AP2-interacting domains of amphiphysin specifically affects the slow component of endocytosis following a 100 ms depolarizing pulse (Jockusch et al., 2005) despite the absence of clathrin-coated vesicles after prolonged stimulation in the wild-type terminals (Paillart et al., 2003). It is possible that clathrin-mediated endocytosis only operates at low [Ca^2+^]_i_, while bulk endocytosis takes over at high [Ca^2+^]_i_ in response to sustained activity. The roles of other proteins involved in clathrin-mediated endocytosis, such as stonin, EPS-15 and intersectin, have not been studied in this system.

Intracellullar dialysis of retinal bipolar neuron terminals has also been used to probe the importance of membrane-remodeling proteins. Endophilin-sequestering peptides or dominant-negative endophilin mutants impair the fast component of endocytosis, while the slow component remains unaffected (Llobet et al., 2011). This result implies that endophilin is specifically involved in the fast component of endocytosis at these synapses. In contrast, dialysis of a dominant-negative amphiphysin construct affects neither the fast nor the slow component (Llobet et al., 2011). Given its localization to the inner plexiform layer (Sherry and Heidelberger, 2005), amphiphysin might still play a role in synaptic vesicle recycling, possibly by mediating vesicle regeneration from bulk endocytic structures. The roles played by synaptojanin and syndapin at retinal bipolar neuron terminals are not clear. Notably, in a synaptojanin-1 knockout zebrafish, the ribbon synapses of cone photoreceptors have fewer synaptic vesicles and accumulate bulk endocytic structures (Epps et al., 2004). In addition, synaptic ribbons become unanchored from active zones (Epps et al., 2004). It is uncertain whether these defects arise because synaptojanin-1 loss disrupts endocytosis or because it generally affects the actin cytoskeleton.

## Calyx of Held

### Anatomical and Functional Overview

**Figure 6:**
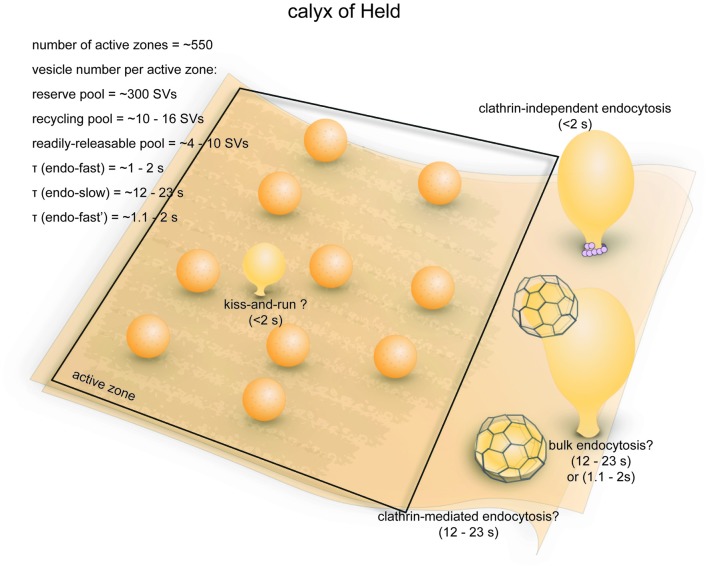
Vesicle pools and endocytic pathways at the calyx of Held. A typical presynaptic bouton of the calyx of Held contains ~550 active zones. At each active zone, 4–10 vesicle constitute the RRP and can fuse in response to a single 20 ms depolarizing pulse. An additional 10–16 vesicles are utilized following 5 min stimulation at 2 Hz, and therefore belong to the recycling pool. Roughly 320 vesicles per active zone are in the reserve pool. Three endocytic pathways exist at the calyx of Held: a fast pathway (*τ*_fast_ ≈ 1–2 s) triggered by single or brief trains of action potentials, a slow pathway (*τ*_slow_ ≈ 12–23 s) triggered by 20 ms depolarizing pulses, and an additional fast pathway (*τ*_fast_ ≈ 1.1–2 s) triggered by repeated 20 ms pulses or high-frequency action potential trains. The fast pathway triggered by single action potentials likely represents ultrafast endocytosis. The slow pathway is likely operated by activity-dependent bulk endocytosis and clathrin-mediated endocytosis. The additional fast pathway induced by intense stimulation might be a dynamin-independent and actin-dependent form of bulk endocytosis.

The calyx of Held is a giant glutamatergic synapse that participates in auditory processing in the mammalian brain stem (Figure 6; Sätzler et al., 2002). The synapse is formed between globular bushy cells (GBCs) and the principal cells of the medial nucleus of the trapezoid body (MNTB; Xue and Mei, 2011). The axon of the GBC branches into 2–4 thick stalks that run along the surface of the MNTB principal cell towards the opposite pole (Rowland et al., 2000). The stalks in turn branch out through thin necks into bulbous swellings of varying sizes (Rowland et al., 2000), covering ~40% of the surface area of the postsynaptic cell body as measured in P9 young rats (Sätzler et al., 2002). At this age, a typical rat calyx of Held forms roughly 550 synaptic contacts with the MNTB principal cell, each with two docked vesicles on average (Sätzler et al., 2002). Another study in P14 rats found ~680 active zones per calyx (Taschenberger et al., 2002). These numbers are relatively close to the electrophysiological estimation of the number of release sites (~640; Meyer et al., 2001). The average surface area of these synaptic contacts is 0.1 μm^3^, comprising a total area of ~55 μm^2^ (Sätzler et al., 2002). Both AMPA and NMDA receptors are present on the postsynaptic side (Joshi and Wang, 2002). The calyx of Held therefore represent a unique model system that is structurally distinct from conventional central synapses of mammals.

Ultrastructural analyses indicate that there are approximately 188,000 synaptic vesicles in an average calyx (de Lange et al., 2003), of which about 70,000 are closely associated with synaptic contacts (Sätzler et al., 2002). The RRP contains 2200–5200 vesicles (4–10 per active zone; 1.3%–2.7% of the total pool; Sakaba and Neher, 2001b; Sun and Wu, 2001). The initial fusion rate of this pool can be more than 300/ms (600/s per active zone), and the entire RRP can be depleted by a 10–20 ms depolarizing (−5 mV) pulse (Sun and Wu, 2001; Wu et al., 2005). The RRP can be further resolved into two heterogeneous populations with fast and slow time constants of fusion (2.4 ms vs. 21 ms, respectively; Sakaba and Neher, 2001a,b). After 5 min of prolonged stimulation at 2 Hz in the presence of FM dye, roughly 6.0% (~11,000) of all vesicles in the calyx are labeled (de Lange et al., 2003). The recycling pool therefore consists of 5800–8800 vesicles (10–16 per active zone). The rest of the vesicles in the calyx (~177,000, or 320 per active zone) presumably constitute the reserve pool. One study proposed an alternate model where the recycling pool makes up ~80% of total vesicles in the calyx (Qiu et al., 2015). Similarly, another study investigated the size of the recycling pool by preventing vesicle re-filling and found that most of the vesicles within a calyx can participate in the vesicle cycle with strong stimulation (20 ms depolarizing pulses at 1 Hz; Xue et al., 2013). However, this might be due to non-physiological strong stimulation that recruit vesicles from the reserve pool, which has been reported by previous studies (de Lange et al., 2003). Thus, it is not clear what percentage of vesicles would participate in synaptic transmission under physiological conditions. Despite the large size of the calyx, the numbers of vesicles in each functional pool per active zone is similar to conventional central synapses (see next section), although the calyx can achieve a much higher fusion rate per active zone.

### Strengths as a Model System

The calyx of Held is a very popular model system for electrophysiological studies on the vesicle cycle. Due to their large size, the presynaptic terminals of calyces in rodent brain slices can be directly patched just like goldfish bipolar neuron terminals (Forsythe, 1994). Changes in membrane capacitance can be measured as a direct readout of exocytosis and endocytosis (Sun et al., 2004). Unlike retinal bipolar neurons, GBCs fire conventional action potentials (Forsythe, 1994). To process auditory signals, the rate of firing ranges from ~50 Hz to ~1 kHz (Rhode, 2008; Kim et al., 2013); this high rate of firing imposes tremendous pressure on the endocytic machinery. The calyx of Held is therefore well-suited to studying synaptic vesicle endocytosis.

### Endocytic Pathways

Activity-induced endocytosis at the calyx of Held has been assessed with membrane capacitance measurement under two general types of stimulation paradigms: mild stimulation by single or short trains of evoked action potentials (Sun et al., 2002), or strong stimulation by 10–20 ms depolarizing pulses (Wu et al., 2005). An early study found that following a single action potential, membrane capacitance decays rapidly at a τ of ~100 ms (Sun et al., 2002), but this result was later found to be due to capacitance artifacts (Wu et al., 2005). More accurate estimates of τ are around 1–2.2 s after correcting for capacitance artifacts (Wu et al., 2005; Renden and von Gersdorff, 2007). The decay slows down with increasing frequency and number of action potentials (down to *τ* = 6.7 s after 10 action potentials at 333 Hz). As in retinal bipolar neurons (von Gersdorff and Matthews, 1994b), the activity-dependent slowing down of endocytosis in the calyx is likely a result of increases in basal [Ca^2+^]_i_ (Wu and Wu, 2014). In response to a single 20 ms depolarizing (−80 to +10 mV) pulse, which depletes the RRP, membrane capacitance recovers slowly at *τ* = 12.0 s (Wu et al., 2005). With 10 RRP-depleting pulses, a Ca^2+^-dependent fast component with *τ* = 1.1–1.4 s appears while a slow component remains (*τ* = 23 s; Wu et al., 2005). A similar activity-dependent fast component (*τ* = 1.3–1.9 s) also emerges with long high-frequency AP trains (Wu et al., 2005). These experiments were performed mostly at room temperature. At physiological temperature, however, even a single 20–30 ms pulse induces a fast endocytic component (*τ* = 2.2 s at P7–10;* τ* = 1.8 s at P14–18; Renden and von Gersdorff, 2007). Furthermore, the overall rate of endocytosis is higher in more mature calyces. Endocytosis at the calyx of Held therefore occurs at a wide range of time scales and is regulated by the level of stimulation (as was first shown in retinal bipolar neuron terminals (von Gersdorff and Matthews, 1994a) and also in other mammalian central synapses (Delvendahl et al., 2016), temperature, as well as developmental stage.

The fast endocytic pathways induced by a single action potential and by repetitive stimuli that deplete RRP are mechanistically different (Figure 6; Wu et al., 2005). They are kinetically similar, as both are faster than clathrin-mediated endocytosis but a little slower than ultrafast endocytosis (100 ms−1 s). The fast endocytosis induced by single action potentials may be mediated by kiss-and-run (Figure 6), the existence of which at calyces has been shown by brief capacitance flickers during stimulation by high K^+^ (He et al., 2006). However, the fast pathway can be accounted for by ultrafast endocytosis as well. The fast pathway is sensitive to actin depletion (Wu et al., 2016) and surface tension (Wu et al., 2017). In addition, this pathway can be completely blocked by a nonhydrolyzable analog of GTP (GTPγS; Yamashita et al., 2005), suggesting that dynamin is likely involved. Fast kinetics and dependence on actin and dynamin are both the hallmarks of ultrafast endocytosis (Watanabe et al., 2013b). In contrast, fast endocytosis triggered by repetitive stimulation is not sensitive to GTPγS (Yamashita et al., 2005), and this component becomes more prominent with intense stimulation (Xu et al., 2008). These results suggest that the fast endocytic pathway induced by intense stimulation is likely a dynamin- and clathrin-independent form of bulk endocytosis (Figure 6), which has been described in another mammalian central synapse (Wu et al., 2014). Thus, despite the similar kinetics displayed by the fast endocytic pathways induced by mild and intense stimulations, the evidence reviewed here imply that these two pathways are likely operated by different mechanisms.

The slow endocytic component evoked by single RRP-depleting pulses, on the other hand, is kinetically consistent with both clathrin-mediated and bulk endocytosis (Figure 6). Following stimulation by high K^+^, large endosome-like vacuoles accumulate in the calyx (de Lange et al., 2003). A large and rapid downward capacitance shift occasionally occurs during slow endocytosis, possibly representing large invaginations being pinched off from the plasma membrane (Wu and Wu, 2007). These results strongly support the involvement of conventional activity-dependent bulk endocytosis. Moreover, a GTP-independent slow endocytic pathway (*τ* = 14 s) is also observed upon repeated RRP-depleting pulses (Xu et al., 2008), suggesting the involvement of dynamin-independent bulk endocytosis (Wu et al., 2014). On the other hand, blocking clathrin-mediated endocytosis slows down the slow capacitance recovery induced by 20 ms depolarizing pulses (Yue et al., 2017). Therefore, clathrin-mediated endocytosis might cooperate with bulk endocytosis in the slow pathway.

### Molecular Requirements

The molecular requirements of endocytosis at the calyx have been studied primarily using chemical probes. GTP-γ-S treatment and intracellular dialysis of dynamin-sequestering protein domains impair fast endocytosis induced by AP-like stimulation and slow endocytosis induced by single RRP-depleting pulses (Yamashita et al., 2005; Xu et al., 2008). As mentioned above, both fast and slow components of endocytosis triggered by repeated strong stimuli seem to be dynamin-independent (Yamashita et al., 2005; Xu et al., 2008). This component might contribute to calyceal response to sound, during which the initial discharge rate of GBCs can reach up to a few kHz (Rhode, 2008). All three endocytic pathways are likely to have actin-dependent and clathrin-independent elements. Knockout of β- and/or γ-actin impairs all forms of endocytosis at calyces (Wu et al., 2016). Compounds that inhibit formin and non-muscle myosin II block slow endocytosis at the calyx (Soykan et al., 2017), indicating the involvement of actin and myosin dynamics. Pitstop 1, which disrupts amphiphysin-clathrin interactions, impairs but does not block slow endocytosis at the calyx (Yue et al., 2017). DNF, a peptide that disrupts amphiphysin-AP2 interactions, has a similar effect (Yue et al., 2017). These results imply that different components of synaptic vesicle endocytosis at the calyx have differential molecular requirements.

## Rodent Hippocampal Synapses

### Anatomical and Functional Overview

**Figure 7:**
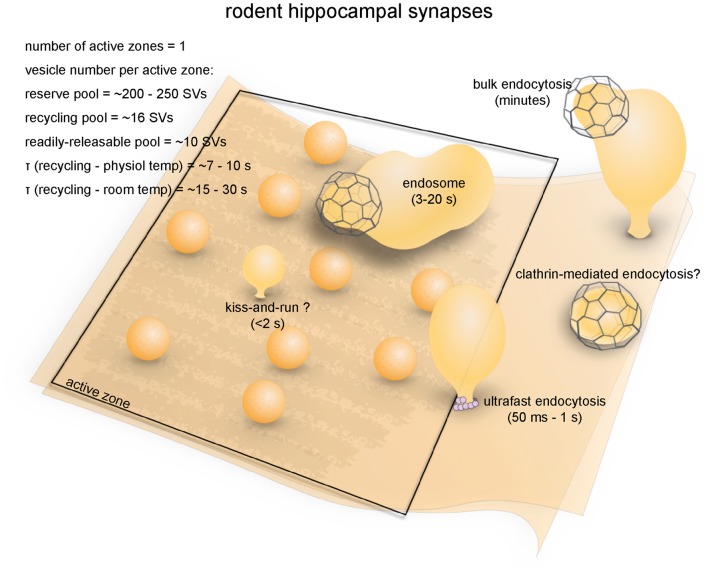
Vesicle pools and endocytic pathways at rodent hippocampal synapses. A conventional small synapse in the hippocampus contains a single active zone, at which about 10 vesicles are docked. These vesicles are in the RRP. An additional 16 vesicles are in the recycling pool, while ~240 are in the reserve pool. Synaptic vesicle recycling occurs on a timescale of 7–10 s at physiological temperatures as measured by optical imaging. This time constant matches the time constant for regeneration of synaptic vesicles through ultrafast endocytosis and subsequent endosomal sorting, but traditionally this pathway is thought to be operated by clathrin-mediated endocytosis. Activity bulk endocytosis occurs in response to intense stimulation and is completed over several minutes. Kiss-and-run has also been proposed but is still debated.

Synapses in the hippocampi of rodents are representative of mammalian central synapses (Figure 7). A pyramidal cell in the rodent hippocampus typically receive around 32,000 synaptic inputs, of which 95% are excitatory (glutamatergic) and 5% are inhibitory (GABAergic; Megías et al., 2001). Excitatory synapses are characterized by a thick post-synaptic density and tend to form on dendritic spines, while inhibitory synapses form on the dendritic shaft, the soma or the axon initial segment (Megías et al., 2001; Harris and Weinberg, 2012). Some GABAergic terminals also contact spines (Chiu et al., 2013). The morphological features of hippocampal synapses vary greatly (Harris and Sultan, 1995). The average volume of excitatory presynaptic boutons is 0.09 μm^3^ (Schikorski and Stevens, 1997), and most have a single active zone with a mean area of 0.039 μm^2^ (Schikorski and Stevens, 1997). The total number of synaptic vesicles per bouton is typically around 270 in brain slices (195 in primary culture; Schikorski and Stevens, 1997). However, in terminals that contact mushroom-type spines, the total number of vesicles can exceed 1000. Although most hippocampal synapses are small, a unique type of giant synapses are formed between mossy fiber axon terminals and CA3 cells. Mossy fiber boutons are 2–13 μm^3^ in volume, each containing on average ~20 active zones and ~16,000 synaptic vesicles (Rollenhagen and Lübke, 2010). For the sake of simplicity, in this review we will focus on the small, conventional type of hippocampal synapses.

Hippocampal synapses have comparatively small vesicle pools (Figure 7). The RRP can be depleted either by a hypertonic shock or by 40 action potentials at 20 Hz (Rosenmund and Stevens, 1996; Murthy and Stevens, 1999). The number of vesicles in the RRP at each bouton correlates well with the number of morphologically docked vesicles, both with a mean of around 10 (~4%; Rosenmund and Stevens, 1996; Schikorski and Stevens, 2001). Based on FM dye loading after 600 action potentials at 10 Hz, the sum of readily releasable and recycling pools is estimated to be around 26 per terminal (Murthy and Stevens, 1999). The recycling pool therefore contains roughly 15 vesicles (~7%), while the reserve pool makes up 80%–90% of the total vesicle pool. Depending on factors such as synapse size and local activity, release probability of excitatory synapses can vary between 0–0.8, skewing heavily towards low values (mean around 0.2–0.4; Slutsky et al., 2004; Branco et al., 2008, 2010). Pyramidal cells in the hippocampus typically fire single action potentials at low frequency (0.1–10 Hz), although some cells can reach a firing frequency of up to 100 Hz (Mizuseki and Buzsáki, 2013). A small fraction of action potentials are fired in brief bursts (inter-spike interval < 8 ms; Mizuseki and Buzsáki, 2013). Thus, the small vesicle pools of hippocampal synapses are likely well-adapted to low *in vivo* firing rates and release frequencies.

### Strengths as a Model System

Rodent hippocampal neurons are a highly versatile model for studying vesicle dynamics. Hippocampal neuronal cultures are easy to prepare and genetic manipulations in cultured neurons are convenient (Kaech and Banker, 2006; Malinow et al., 2010). Alternatively, acute or organotypic slices of hippocampus can be prepared to study synaptic processes in relatively intact neural circuits (Humpel, 2015). In addition, a massive number of genetic modified mouse lines (including conditional or tissue-specific knock-outs and knock-ins) are available (Cho et al., 2009), greatly expediting work on the molecular machinery of vesicle recycling. The principal cell type in the hippocampus, the pyramidal neuron, is found throughout the entire forebrain, including in the cerebral cortex and the amygdala (Spruston, 2008). Therefore, information derived from hippocampus would likely be applicable to many brain regions.

### Endocytic Pathways

Early studies established clathrin-mediated endocytosis as a primary pathway for synaptic vesicle recycling in mammalian central synapses (Figure 7). Biochemically purified coated vesicles have major components of synaptic vesicles (Maycox et al., 1992), suggesting that synaptic vesicles pass through a clathrin-mediated process. Reconstitution assays suggest that clathrin and AP2 purified from brain lysates are sufficient to generate vesicles from liposomes (Takei et al., 1996, 1998). At room temperature, optical imaging also detects slow endocytosis with α varying from 15 s to 30 s (Heuser and Reese, 1981; Ryan et al., 1993, 1996; Pyle et al., 2000; Granseth et al., 2006, 2007; Balaji and Ryan, 2007; Balaji et al., 2008; Granseth and Lagnado, 2008; Hua et al., 2011; Schikorski, 2014; Villarreal et al., 2017). At physiological temperature (35–37°C), this slow pathway becomes faster, with τ ≈ 7–10 s (Granseth and Lagnado, 2008; Armbruster et al., 2013). The kinetics of endocytosis are similar to the morphological appearance of clathrin-coated pits in frog neuromuscular junctions (Heuser and Reese, 1981). This endocytic pathway is sensitive to perturbing clathrin and clathrin-associated proteins (Granseth et al., 2006, 2007), further supporting that the slow pathway is clathrin-mediated.

However, recent studies have challenged this original view and suggested the presence of clathrin-independent pathways at mammalian central synapses (Figure 7). Following a single optical stimulus in neurons expressing channelrhodopsin, ultrafast endocytosis is observed at the region just outside of the active zone (Watanabe et al., 2013b). This endocytic pathway initiates within 50 ms of stimulation and carries on for ~1 s. Ultrafast endocytosis is compensatory, as the amount of membrane internalized on average equals the amount exocytosed (Watanabe et al., 2013b). The endocytic vesicles are then delivered to endosomes, from which synaptic vesicles are regenerated by clathrin (Watanabe et al., 2014). The whole cycle takes approximately 10–20 s, which is similar to the time constant measured by optical imaging at 37°C (Balaji et al., 2008; Granseth and Lagnado, 2008). Clathrin-independent, temperature-sensitive rapid endocytosis (*τ* = ~1 s) is also found at hippocampal mossy fiber synapses following a single action potential (Delvendahl et al., 2016). This pathway may be similar to fast compensatory endocytosis found at retinal bipolar neuron ribbon synapses (von Gersdorff and Matthews, 1994a). However, given its sensitivity to actin perturbation (Delvendahl et al., 2016), this pathway likely represents ultrafast endocytosis. A more recent study using rapid acid quenching of pHluorin suggests that all endocytic events following 10 Hz stimulation are formin-dependent, clathrin-independent, and this actin-dependent pathway occurs on multiple time-scales (*τ*_fast_ = 760 ms and *τ*_slow_ = 26 s; Soykan et al., 2017). When neurons subjected to high-frequency stimulation or high K^+^, bulk membrane is internalized via a clathrin-independent mechanism (Clayton and Cousin, 2009). Synaptic vesicles are then regenerated from the internalized cisternae. Collectively, these studies suggest that synaptic vesicle endocytosis at mammalian central synapses is clathrin-independent for both mild and strong stimulations.

How do we reconcile these contradictory findings? One possibility is the temperature at which experiments are performed. Earlier studies were often performed at room temperature, where ultrafast endocytosis is blocked and clathrin-mediated endocytosis takes over in its place (Watanabe et al., 2014). Indeed, studies that perform similar experiments at physiological temperature oftentimes found clathrin-independent forms of synaptic vesicle endocytosis at mammalian central synapses (Watanabe et al., 2013b; Delvendahl et al., 2016; Soykan et al., 2017). However, one study showed that even at 27°C, knocking down AP2 and CHC did not block synaptic vesicle endocytosis, but only impaired endosomal budding (Kononenko et al., 2014). In addition, a few studies have also found a fast component with α on the scale of 1–3 s (Pyle et al., 2000; Sara et al., 2002) even at room temperature. Similarly, a newly published study using single-vesicle pHluorin imaging found that a fraction of activity-induced endocytic events is ultrafast (mean duration = 200–300 ms) at 24°C (Chanaday and Kavalali, 2018). However, it is questionable whether these fast endocytic pathways at room temperature are attributed to the same clathrin-independent endocytosis observed at the physiological temperature or it represents kiss-and-run endocytosis. The other possibility is that the endocytic defects found in earlier clathrin/AP-2 knockdown studies were due to the use of lipofectamine for transfection, which may have impaired synaptic vesicle cycle independent of the knockdown (Kononenko et al., 2014; Soykan et al., 2017). Alternatively, both clathrin-dependent and -independent endocytic mechanisms may operate depending on the stimulation frequency, perhaps by calcium-dependent toggling between different modes of endocytosis (Kononenko and Haucke, 2015). It is possible that fast clathrin-independent endocytosis is responsible for the reuptake of vesicular membrane and some of the vesicular proteins immediately after synaptic vesicle fusion, while clathrin-mediated endocytosis is needed to fully recycle all remaining vesicular proteins on a slower timescale. This is supported by the finding that endocytosis of vesicular proteins operates on multiple time scales (Zhu et al., 2009; Soykan et al., 2017; Chanaday and Kavalali, 2018).

Furthermore, an alternative fast pathway, kiss-and-run, has been found at mammalian central synapses (Figure 7; Gandhi and Stevens, 2003; Zhang et al., 2009), although whether this is a dominant mechanism is still debated (He and Wu, 2007). One study used quantum dots to assay the transient opening and closing of fusion pores during intense stimulation by K^+^ (Zhang et al., 2009). However, the large size of the quantum dots might have affected the biophysics of fusion pore opening and vesicle collapse (Dittman and Ryan, 2009). Another study found a fast component of vesicle reacidification (430–860 ms) using single-synapse pHluorin imaging (Gandhi and Stevens, 2003). Indeed, this component is faster than the full time course of vesicle regeneration described for ultrafast endocytosis (3–10 s; Watanabe et al., 2014) and seemingly could only be attributed to kiss-and-run. However, it is possible that the rapid decline in pHluroin signal in this case is due to the diffusion of pHluroin out of the small regions-of-interest following full collapse (Granseth et al., 2006). Thus, more work is needed to determine the importance of kiss-and-run at hippocampal synapses.

### Molecular Requirements

Like in other systems, dynamin is critically important for synaptic vesicle endocytosis at hippocampal synapses. The functions of dynamin are regulated by post-translational modifications. The proline-rich domain of dynamin 1 is phosphorylated at S774 and S778 at rest by either Cdk5 (Tomizawa et al., 2003) or GSK3 (Clayton et al., 2010); calcium-induced dephosphorylation of these sites through calmodulin/calcineurin accelerates the kinetics of endocytosis (Sankaranarayanan and Ryan, 2001; Sun et al., 2010; Xue et al., 2011). Dynasore, which inhibits the GTPase activity of dynamin, completely blocks clathrin-mediated endocytosis at room temperature (Newton et al., 2006) and ultrafast endocytosis at physiological temperature (Watanabe et al., 2013b). However, dynasore has off-target effects (Park et al., 2013), and thus whether dynamin is involved in ultrafast endocytosis at mammalian central synapses is still questionable. It is worth noting that ultrafast endocytosis at *C. elegans* neuromuscular junctions is dynamin-dependent as demonstrated in the temperature-sensitive mutant of *dyn-1* (Watanabe et al., 2013a). Dynamin knock-out studies found that clathrin-coated pits are stalled on membrane involutions following high K^+^ application at room temperature, suggesting that dynamin is involved in clathrin-mediated endocytosis. Similarly, bulk endocytosis also requires dynamin function, although a dynamin-independent form of bulk endocytosis may also exist (Wu et al., 2014). Nevertheless, dynamin is fundamental in synaptic vesicle recycling at mammalian central synapses.

Clathrin is also essential in synaptic vesicle recovery in these terminals. Knockdown of CHC fully blocks recycling (as measured by pHluorin) following mild stimulation (4 APs, 20 Hz) at room temperature, but only partially blocks recycling processes triggered by stronger stimulation (40 APs, 20 Hz; Granseth et al., 2006). This weaker effect is likely caused by an inhibition of endosomal budding, as recent articles suggest that clathrin functions at endosomes (Watanabe et al., 2014). Similarly, depleting other clathrin-associated proteins such as AP180, AP1–3, Stonin-2, intersectin and Eps15 also causes defects in synaptic vesicle recycling (Granseth et al., 2006; Kim and Ryan, 2009; Thomas et al., 2009; Glyvuk et al., 2010; Willox and Royle, 2012; Li et al., 2017), suggesting these molecules are fundamental to regenerating vesicles. Endophilin and synaptojanin are also involved in synaptic vesicle recycling by removing clathrin coats from synaptic vesicles (Chen et al., 2003; Milosevic et al., 2011; Zhang et al., 2012, 2015). Thus, clathrin and clathrin-associated proteins play essential roles, but whether they all function at the plasma membrane is under debate.

## Summary and Perspectives

In summary, all seven model synapses reviewed here share common mechanisms and molecular machinery for synaptic vesicle endocytosis. Multiple modes of endocytosis seem to be present at each type of synapses, and different preferences for these modes might underlie quantitative differences in recycling kinetics and differences in molecular requirements. By reviewing a huge body of literature regarding synaptic vesicle recycling, four factors have become apparent. First, the fast form of endocytosis is only observed by capacitance measurements in large synaptic boutons (von Gersdorff and Matthews, 1994b; Sun et al., 2002; Wu et al., 2005) or by time-resolved electron microscopy (slam-freezing and flash-and-freeze) using brief stimuli (Miller and Heuser, 1984; Watanabe et al., 2013a,b). Optical based assays are typically not direct measurements of endocytosis, since the change in pHluorin signal is partially limited by vesicle acidification, and FM dye wash-in requires time. Thus, the fast component is not always apparent from traces, although it has been observed in some studies (Leitz and Kavalali, 2011; Soykan et al., 2017). Second, fast clathrin-independent endocytosis (ultrafast endocytosis or fast compensatory endocytosis) tend to predominate following brief stimuli that are closer to the physiological activity patterns of the neurons (von Gersdorff and Matthews, 1994a; Sun et al., 2002; Watanabe et al., 2013a,b; Delvendahl et al., 2016). In contrast, slow endocytosis (clathrin-mediated endocytosis or bulk endocytosis) tend to occur when the number of stimuli applied far exceeds what these terminals would experience *in vivo*, although notable exceptions do exist (Xu et al., 2008). Third, activity-dependent bulk endocytosis is induced when the recycling pool is depleted and the reserve pool is mobilized (Table 2). This pool of vesicles is not used under physiological conditions (Rizzoli and Betz, 2005). Thus, bulk endocytosis likely provides an emergency backup program to recover functional pools during periods of intense activity. In these conditions, the recovery of vesicle pools requires minutes (Richards et al., 2000; Clayton and Cousin, 2009)—much longer than typical time constants for synaptic vesicle recycling (seconds). Fourth, clathrin-coated pits are observed in almost all of these synapses. Recent studies indicate that fast clathrin-independent endocytosis has a limited capacity for recovery of vesicle proteins (Soykan et al., 2017), and thus the excess proteins on the plasma membrane may trigger clathrin-mediated endocytosis as a complementary mechanism for retrieving synaptic proteins. Our general conclusion based on these findings is that every type of neuron is equipped with various endocytic modes and may switch between them based on environment and activity levels. Further studies are needed to test this hypothesis.

**Table 2:** Activity-dependent bulk endocytosis occurs when the recycling pool is depleted and the reserve pool is mobilized.

	Stimulation protocol for bulk endocytosis	# of vesicles fused during stimulation	Folds of recycling pool consumed	Percentage of reserve pool mobilized
Frog neuromuscular junction	30 Hz, 1 min (Richards et al., 2000)	~200,000 (based on HRP labeling of vesicles, Rizzoli and Betz, 2004)	2.7–6.6 x	29%–36%
*C. elegans* neuromuscular junction	30 s non-pulsed laser stimulation (Kittelmann et al., 2013)	?	?	Presumably ~100%
*Drosophila* neuromuscular junction	1600 APs at 80 Hz (Yao et al., 2017)	~20,000 (assuming 1000/s steady-state fusion rate, Delgado et al., 2000)	1.8 x	12%
Lamprey reticulospinal giant synapses	20 Hz, 20 min (Gad et al., 1998)	~7200 (assuming a constant *Pr* of 0.3, Photowala et al., 2006)	1.5 × (assuming a total vesicle # of 12,000)	33%
Retinal bipolar neuron terminal	1 min depolarization at 2.5 mM Ca^2+^ (Holt et al., 2003)	~228,000 (assuming a fusion rate of 3800/s, Lagnado et al., 1996)	51 x	25%–46%
Calyx of Held	10 × 20 ms depolarizing pulse at 10 Hz (Wu and Wu, 2007)	~16,300 (assuming 1278 fF jump after stimulation, Wu et al., 2005; and 12.7 quanta per fF, Sun and Wu, 2001)	1.5 x	3%
Hippocampal synapses	200 APs at 80 Hz (Wenzel et al., 2012)	~80 (assuming a constant *Pr* of 0.4, Branco et al., 2010)	3.2 x	22%–31%

*Numbers of vesicles released by protocols that induce activity-dependent bulk endocytosis are estimated based on the percentage of HRP-labeled vesicles (as in frog muscular junctions), the steady-state release rate (as in *Drosophila* neuromuscular junctions and goldfish retinal bipolar neuron terminals), the total increase in membrane capacitance (as in the calyx of Held), or the average *Pr* at a given type of synapse (as in lamprey giant synapses and rodent hippocampal synapses). Percentage mobilization of the reserve pool is calculated based on the assumption that the reserve pool starts to be mobilized after the entire recycling pool has been depleted once. Note that the stimulation protocols listed here might not represent the minimum amount of stimulation required to triggered activity-dependent bulk endocytosis*.

For the purposes of the review, we have treated three clathrin-independent mechanisms (ultrafast endocytosis, fast compensatory endocytosis, and activity-dependent bulk endocytosis) as separate mechanisms based on their kinetics and molecular requirements, but the most important emerging concept in the field is that unlike the classical view, synaptic vesicle endocytosis is clathrin-independent. Thus, future investigation is warranted to test whether this concept is valid and if so, tease out why clathrin does not function at the plasma membrane under the physiological conditions and under what circumstances is clathrin required at the plasma membrane. In addition, whether all three clathrin-independent endocytic pathways share general mechanisms must be determined.

In order to determine the mechanism of synaptic vesicle endocytosis, a combination of multiple experimental approaches is needed, since each approach has distinct strengths and weaknesses. For example, the time-resolved electron microscopy approach is sensitive to events faster than a second, but less sensitive to slow events. In addition, only a single time point can be observed for a given sample due to the nature of electron microscopy, and thus many synapses must be reconstructed (Watanabe, 2016). In contrast, pHluorin-based assays can track vesicle proteins in real time at live synapses. However, tens of action potentials are typically applied due to poor signal-to-noise ratios. Furthermore, this approach has difficulty distinguishing events at the plasma membrane (e.g., protein diffusion and endocytosis) from intracellular trafficking events such as vesicle acidification and endosomal sorting (Kavalali and Jorgensen, 2014). In addition, vesicle proteins tagged with pHluorin are expressed with untagged wild-type copies, and thus, the number of tagged proteins in each vesicle is likely variable. Given the requirement for post-endocytic trafficking to endosomes and endosomal sorting (Kononenko et al., 2014; Watanabe et al., 2014), it is also worth considering when reacidification initiates during synaptic vesicle recycling and whether each endocytic vesicle would contain all vesicle proteins (i.e., synaptobrevin, synaptophysin, v-ATPase, v-Glut1). The lack of v-ATPase in endocytic vesicles would delay reacidification until endocytic vesicles are delivered to endosomes, giving rise to the appearance that proteins are on the surface since signals would not quench. Nevertheless, recent advances in single-stimulus pHluorin imaging and fast acid quenching might help overcome these drawbacks (Balaji and Ryan, 2007; Zhu et al., 2009; Chanaday and Kavalali, 2018). Finally, capacitance recordings measure membrane flux at sub-millisecond temporal resolution but cannot track endocytic vesicles in the cytoplasm. It is also only possible at synaptic terminals that are large enough to patch (von Gersdorff and Matthews, 1999; Sun et al., 2004). These terminals typically have multiple active zones, and therefore the exact locations of endocytic events cannot be revealed by this approach. Thus, a combination of these techniques is needed to fully understand the synaptic vesicle cycle.

The molecular requirements of synaptic vesicle recycling in different model systems are, as expected, similar. Dynamin, for example, is almost invariably required for the major endocytic pathways at all seven types of synapses, the only exceptions are dynamin-independent forms of endocytosis found at the calyx of Held and cultured cortical neurons during highly intense stimulation (Xu et al., 2008; Wu et al., 2014). However, it is unknown whether this form of endocytosis is physiologically relevant. Other proteins, such as those commonly associated with clathrin, are required either for endocytosis or for the regeneration of vesicles from endosomal intermediates. Some slight differences do exist among the model systems concerning the exact role of certain proteins. For example, amphiphysin contributes to endocytosis at *Drosophila* neuromuscular junctions and lamprey reticulospinal giant synapses (Takei et al., 1999; Evergren et al., 2004), but is only involved in vesicle regeneration at *C. elegans* neuromuscular junctions (Pant et al., 2009). This is probably because *C. elegans* neuromuscular junctions rely less on clathrin-mediated endocytosis than the other two systems. Thus, differences in molecular requirements among model systems is likely due to their differential preference for modes of endocytosis and vesicle regeneration.

Finally, the potential diversity of synaptic vesicle endocytosis cannot be fully encompassed by the model systems reviewed here. All seven model systems reviewed here represent classic fast neurotransmission. However, synaptic vesicle endocytosis at slow-releasing synapses might use different mechanisms. For instance, kiss-and-run, which is not prominently featured in the model systems reviewed here, is a major mechanism underlying exo/endocytosis in chromaffin cells (Perrais et al., 2004; Elhamdani et al., 2006; Chiang et al., 2014) and possibly at midbrain dopaminergic terminals (Staal et al., 2004). Recent studies combining STED imaging and capacitance measurements are beginning to elucidate the detailed cellular and molecular mechanism of vesicle dynamics in these cells. For example, one study in chromaffin cells found seven different modes of membrane dynamics during kiss-and-run fusion (Chiang et al., 2014). Another study in the same model system showed that actin dynamics regulates different modes of vesicle fusion through membrane tension (Wen et al., 2016). Nevertheless, vesicle dynamics in other slow neurosecretory and neuromodulatory systems (serotonergic, neuropeptides etc.) have not been systematically studied and therefore warrant further investigation. Second, hippocampal synapses and the calyx of Held, both of which glutamatergic and excitatory, have been treated as representatives of mammalian central synapses. However, there might be quantitative differences between synaptic vesicle endocytosis at excitatory synapses and inhibitory synapses in terms of kinetics, regulation, as well as molecular requirements (Egashira et al., 2016). Future studies could potentially illuminate these differences and their impact on circuit dynamics.

## Author Contributions

QG and SW contributed equally to the work.

## Conflict of Interest Statement

The authors declare that the research was conducted in the absence of any commercial or financial relationships that could be construed as a potential conflict of interest.
